# Towards metaphors for quantum computational thinking

**DOI:** 10.1140/epjqt/s40507-026-00502-1

**Published:** 2026-04-03

**Authors:** Caiseal Beardow, Pieter Jan Stappers

**Affiliations:** Faculty of Industrial Design Engineering, Delft University of Technology, Landbergstraat 15, Delft, 2628CE Zuid-Holland The Netherlands

**Keywords:** Quantum computing, Metaphors, Computational thinking, Quantum education, Expert rating

## Abstract

In recent years, the quantum computing industry has seen significant investment and growth. However, this burgeoning industry faces a persistent labour gap: individuals with computing expertise, an understanding of quantum principles, and the ability to apply these principles to computing practices, are in increasing demand, but finding such individuals is proving challenging. We frame this problem as one of education, arguing that a computing-centred approach to learning about quantum computing is needed, and that the notion of computational thinking can help to define appropriate learning goals and outcomes in this context. We propose that metaphors can be an effective pedagogical tool in supporting the development of ‘quantum computational thinking’.

In this paper, we present our efforts to gather and assess a collection of metaphors that are currently used to teach quantum computing concepts. We describe a series of interviews with quantum computing experts in which we aimed to elicit such metaphors, and document our process of metaphor identification and metaphorical model synthesis. We subsequently assess these metaphors and models using both numerical rating data from experts and our own qualitative analysis.

Informed by our findings, we suggest ways of developing metaphors that better support quantum computational thinking: emphasising target concepts’ computational roles, acknowledging connections between concepts, and balancing procedural narratives with embodied, tangible imagery.

## Introduction

Quantum computing (QC) is an emerging computing paradigm that uses quantum mechanical principles to perform computational tasks [1]. In quantum computing, information is encoded as quantum states, which behave differently to the binary information that digital (referred to as ‘classical’) computing systems employ. These differences in behaviour give quantum computing the potential to efficiently solve certain types of complex problems—some of which are highly challenging, or even intractable, using current computing paradigms [2].

Although quantum computing is presently a largely experimental field [3, 4], in recent years it has received significant investment from both commercial and governmental stakeholders [5, 6]. In tandem, industrial applications are beginning to materialise [7]—and with them, workforce needs [8–11] that are expanding outside the physics community in which the technology originates. As such, knowledge and skills from other disciplines are becoming critical to the progress of quantum computing.

### The need for computing in quantum computing

In recent years, quantum computing companies have expressed a growing need for employees with computer science backgrounds. Computer science-related skills and practices, such as software engineering, algorithm design, and general programming, are increasingly in demand [8, 10] as companies seek to develop more robust and scalable quantum computing systems. At the same time, given the differences between quantum and classical (i.e. binary) information, these employers are looking for a degree of ‘quantum awareness’ in their prospective employees: a sufficient understanding of quantum mechanical concepts to recognise and account for them in computing work [10].

Finding such individuals, however, is proving to be challenging. A number of quantum workforce development surveys [7–10] have identified a persistent—and, according to some industry stakeholders, worsening [10]—lack of disciplinary diversity in the talent pool. Part of this issue may stem from the ways in which quantum computing is typically taught: mostly at university level, in physics programmes [8, 9, 11], and with a resulting physics-led approach to course content [12, 13]. Despite ongoing efforts to deliver interdisciplinary (higher) education on quantum computing (as described in works such as [13–15]), explicitly computing-focused education is relatively scarce [12, 16, 17], and knowledge exchange between quantum computing and computer science communities remains limited [18]. Best practices for delivering computing-focused education on this topic, [12, 19], including appropriate learning goals and outcomes [20], are thus currently unclear.

### Towards quantum computational thinking

Computing educators have begun to explore how quantum computing might be situated within computer science curricula, using framings such as probabilistic computing [12, 21, 22], logic gates [23, 24] and assembly language programming [24]. However, few of these interventions explicitly relate their quantum-specific content to computing skills or practices [12, 17], with key quantum computing concepts remaining theoretical and abstract. Consequently, the accessibility and utility of these interventions for computing-centred learners is likely to be limited [17, 19].

Some recent works propose alternative approaches, focusing on problem-solving [12, 17] and using the design of algorithms [17] and software architectures [25] as a means of demonstrating quantum concepts. Such approaches, in which emphasis is placed on solving problems with (quantum) computing, intersect with the notion of computational thinking. Computational thinking (CT) refers to the cognitive skills people use to frame problems in terms of how they might be solved with computing [26, 27]. CT has been applied as a pedagogical framework in a number of subject areas, primarily in computer science [26–28] but additionally in mathematics [29, 30] and the natural sciences [30], including physics [31, 32]. Although CT is not necessarily synonymous with programming [33], many educational activities designed to support CT skills development involve (pseudo-)code or other means of “designing systems” that draw upon “the concepts fundamental to computer science” [26]. In this way, CT situates subject-specific knowledge within computing concepts and practices, helping learners to articulate connections between the two.

In light of the aforementioned need for quantum-aware computing specialists and educational materials with which to train them, CT appears to be a promising approach. Using CT skills development to structure learning activities and outcomes could help to connect quantum-specific knowledge—the quantum mechanical concepts that form the core of existing quantum computing curricula—to computing concepts and practices. Some initial explorations in this space have been conducted in recent years: Angara and colleagues advocate for and demonstrate the integration of classical and quantum computing concepts within a CT skills development programme for secondary education [34]; Nita and colleagues have developed an educational game titled *Quantum Odyssey*, with the explicit goal of supporting what the authors term “quantum computational thinking”, also citing quantum computing workforce needs as a motivating factor [35]. However, given the nascency of this educational space, the question of how best to design and deliver such education is an open one.

### Supporting quantum computational thinking through metaphors

Recent scholarship highlights metaphors as a critical aspect of designing educational materials for CT skills development [36–39]. Metaphors are described in Lakoff and Johnson’s oft-cited definition as a means of “understanding and experiencing one thing in terms of another” [40], achieved through “general mappings across conceptual domains” [41]. Such mappings are made between *target concepts* and *source domains*: a novel concept one wishes to understand, and a familiar realm of experience from which metaphorical imagery is drawn [40]. When there are a number of metaphors with the same target concept and overarching source domain, they can be grouped into *metaphorical models*:^1^ systems of complementary metaphors that structure our ideas around a given concept [40, 42, 43]. Metaphors and metaphorical models not only aid in understanding novel concepts, but in subsequently making inferences about those concepts, settings goals related to them, and deciding how to act [40, 43].

For example, when learning how to write in an academic setting, we are taught to *construct arguments*. We learn that *arguments* should be *strong* so that they *hold up* under scrutiny, which is achieved by providing sufficient *support* for them. All of these metaphors frame the target concept of an *argument* using the source domain of *construction*. We can thus group them under a metaphorical model, such as *arguments are constructions* or, as Lakoff and Johnson put it, *arguments are buildings* [40]. This metaphorical model (and the metaphors it contains) helps us to understand the concept of an argument, what we should aim for when writing one, and how we should go about doing so.

As such, metaphors are a well-established topic of discussion in education research. They are commonly used in computer science education [44–46], and are in fact evident in many aspects of computer science practice and terminology [47, 48]. Metaphors have also been employed quite extensively in physics education, with documented success in helping learners to grasp highly abstract and complex concepts [49–51]. In quantum physics education, some work argues that metaphors are abundant in the language of instruction, and therefore critical to students’ understanding of quantum mechanics [52]. However, the centrality of metaphors in novice learners’ conceptual development can incur risks: Bitzenbauer and Ubben propose that if novice learners primarily base their mental models of physics concepts on highly visual metaphors, they may misinterpret those metaphors as literal representations of the concepts in question, thus limiting conceptual understanding [53]. Additionally, Brookes and Etkina argue that because metaphors are so critical in building quantum mechanical understanding, educators must be especially cognisant of how such metaphors could be misinterpreted by students and impart an unstable basis for further learning [52]. Despite these risks, other works suggest that metaphors can be a valuable tool in quantum physics education. For example, Pospiech reports positive educator responses to metaphor-based explanations for the concepts of uncertainty and entanglement, particularly regarding the metaphors’ potential to visualise and clarify abstract formalisms [54]. Faletič further demonstrates that metaphors and analogies can effectively support novice students in navigating complex concepts such as superposition and wave-function collapse–particularly when students are actively involved in analogy development [55].

Additionally, metaphors are central to recent work exploring the integration of CT and quantum computing: Angara et al. [34] report a number of educational activities that employ spatial metaphors; likewise, Nita et al. [35] make extensive use of visual metaphors in their educational game *Quantum Odyssey*, arguing that such visualisations are central to the game’s efficacy in teaching both quantum concepts and CT skills. Metaphors might then be used as a starting point for developing CT-informed educational materials for quantum computing. However, there are almost no documented collections of metaphors specifically for quantum computing concepts; the work of Hilkamo and Granqvist [56] is to our knowledge the only such example, and whilst the authors offer a range of metaphors, they do not assess these metaphors’ quality. This leads us to ask how, given our pedagogical and computing-centred framing, we should assess metaphors for quantum computing concepts.

One relatively common approach to assessing metaphors is the definition and numerical rating of various *dimensions*. Katz et al. [57] define ten dimensions (referred to as “norms” by the authors) of linguistic metaphor quality, since validated with a variety of populations [58]. These dimensions include *comprehensibility*, *ease of interpretation* and *semantic relatedness*, amongst others. Discussions of metaphor in the science education literature show some overlap with these dimensions, but also highlight other considerations that are specific to the use of metaphor in teaching and learning scientific concepts. For example, distinctions between teacher (the conveyor of the metaphor) and student (the receiver of the metaphor) are drawn when discussing metaphors’ comprehensibility and ease of interpretation, recognising the influence of prior knowledge of a target concept on metaphor processing [51, 59]. Likewise, the (mis)alignment between a student’s interpretation of a metaphor, their subsequent conclusions regarding that metaphor’s target concept, and the intended conclusions as defined by the teacher, are considered integral to the metaphor’s pedagogical quality [51, 60].

A number of works identify the use of *embodied* source domains as a further critical factor. Niebert et al. [51] define an embodied source domain as relating learners’ “direct experience” to their “physical and social environment”—in other words, metaphorical imagery that is situated in the real world and that is relatable for the learner. They, and other science education scholars, argue that embodied source domains are critical to metaphors’ success in conveying scientific concepts [51, 61]. The importance of embodiment in metaphor has been similarly explored in the computer science education literature [62–64], having more recently been linked to supporting computational thinking skills [36, 38, 39]. Further, a number of works assert a further distinction between metaphors in science and computer science, emphasising the need for metaphors to incorporate actions and observable consequences in order to be computationally relevant [47, 63, 65].

In sum, the pedagogical quality of metaphors can be assessed through a combination of more general and discipline-specific factors, implemented as dimensions for numerical ratings. Returning to the case of quantum computational thinking, if existing metaphors for quantum computing concepts were assessed in such a manner—using dimensions pertaining not only to metaphors in general, or science, but also to computer science and CT—then this might provide an actionable starting point for the development of effective, accessible educational materials.

### Research questions

Summarising the motivations and related works discussed above, we arrive at the following research questions, to be explored in this paper:

1. Which metaphors do quantum computing experts use to describe and explain quantum computing concepts?
2. To what extent do these metaphors support quantum computing knowledge acquisition, according to quantum computing experts?
3. What metaphorical models, if any, might govern these metaphors?
4. To what extent could these metaphors and metaphorical models support computer science practices and computational thinking skills?
5. How might the collected metaphors and metaphorical models be iterated on to better support quantum computational thinking?

In the following sections, we first describe our methods of collecting metaphors from quantum computing experts, assessing their quality, and synthesising metaphorical models. We then present our collected metaphors and models, alongside quality assessment data and our interpretations thereof. We go on to analyse the metaphors and models as pedagogical tools from a computing-centred perspective, reflecting on their potential to support learners in developing quantum computational thinking, and suggesting how they could be redesigned to do so more effectively.

## Method

**Figure 1:**
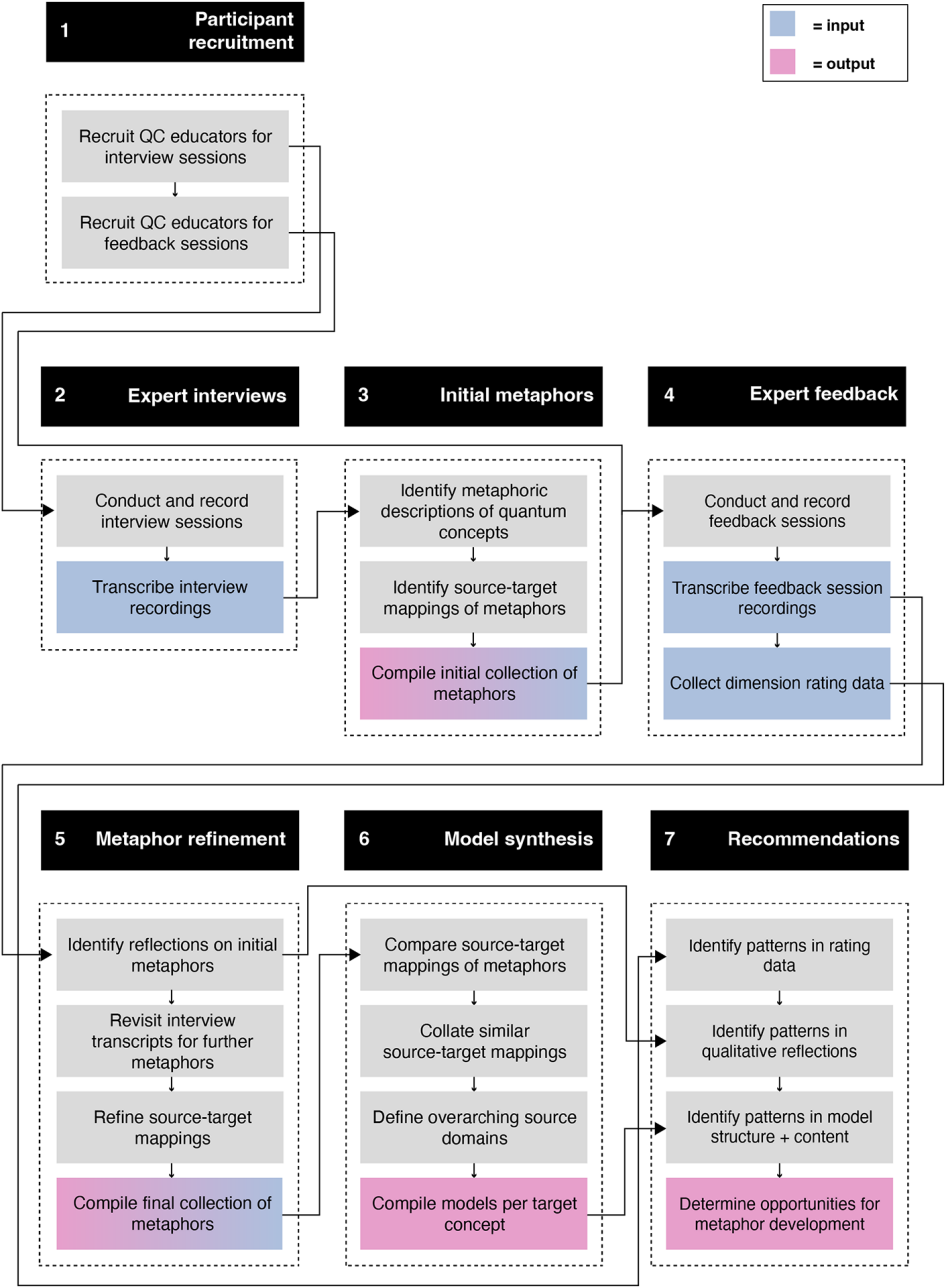
Diagrammatic overview of our method for metaphor collection and refinement. Colour coding indicates where data gathered in one step is used as input for another, or presented as research output

We collected and analysed metaphors for quantum computing concepts according to Schmitt’s Systematic Metaphor Analysis framework [43], first gathering raw data in the form of interview transcripts with quantum computing experts, performing an initial round of metaphor identification, subsequently inviting quantitative and qualitative input from further quantum computing and science communication experts, and finally synthesising our final set of metaphors and associated metaphorical models. Our method is summarised in Fig. 1, which illustrates the various steps and sub-steps involved, including data inputs and outputs. We explain each step in detail in the following sections.

### Participant recruitment

We recruited two groups of expert participants for our study. The first group were recruited to take part in semi-structured interviews, during which we intended to gather metaphors used by the participants to explain quantum computing concepts. The second group were recruited to assess the quality of the metaphors we collected from the first group.

For the first group, we approached participants through our professional networks via email inquiry, with the following recruitment criteria: (I) current involvement in quantum computing-related research or industry; (II) current involvement in quantum computing-related education. These criteria were formulated to target individuals with both deep knowledge of quantum computing concepts and experience in communicating them to others aiming to engage with quantum computing.

For the second group, we again approached participants through our professional networks, with the recruitment criteria of: (I) current involvement in quantum computing-related research or industry; (II) current involvement in quantum computing-related education or science communication. Although some of the participants from our first set of interviews were included in our recruitment pool, we consciously sought additional participants with whom we had not previously spoken. Our recruitment criteria and process thus aimed to target individuals with relevant expertise whilst broadening the represented perspectives on both quantum computing and metaphor.

### Initial metaphor collection

With our first group of participants, we conducted individual exploratory interviews in combination with ZMET [66]: a validated metaphor-elicitation technique that makes use of participants’ own visual materials pertaining to a given topic [67, 68]. Prior to the interviews, we asked each participant to prepare an example of visual material that they considered to be representative of quantum computing. During the interviews, we discussed each participant’s visual materials with them, asking them to identify and explain the quantum computing concepts that they felt their materials conveyed. Following the discussion of the participants’ materials, we asked the participants explicitly to share any further metaphors they had encountered pertaining to the concepts they had identified. All interviews were audio recorded, transcribed and anonymised in line with our institution’s Human Research Ethics policy [69], and participants’ written, informed consent for audio recording, data processing, and publication of anonymised data was obtained prior to the commencement of each interview session.

Following the expert interviews, we conducted an initial round of metaphor identification. We reviewed the interview transcripts and noted any passages containing metaphorical language (according to Schmitt’s criteria [43]) related to quantum computing concepts, then copied these passages verbatim to a repository document, assigning each an alphanumeric code for ease of discussion amongst the research team. We then interpreted the conceptual mappings of the passages, comparing and synthesising our interpretations to arrive at a likely target concept and source domain for each.

### Metaphor assessment

Having produced an initial set of metaphors, we conducted feedback sessions with our second group of participants. During these sessions, we began by asking our participants to reflect on our proposed conceptual mappings for the initial metaphor collection, making note of any alternative or additional mappings they suggested. We then asked our participants to assign each metaphor with a rating from 1 to 5 (1 being minimal, 5 being maximal) across six dimensions of metaphor quality. We formulated these dimensions using various literature sources in which metaphors were assessed for both general and pedagogical quality (see Sect. 1.3 for further discussion). Dimensions of general quality were derived from Katz et al. [57]’s metaphoric norms, whilst pedagogical dimensions were compiled from prior assessments in science education [49, 51, 70], computer science [47, 63, 65, 71], and computational thinking [36–38, 72].

We define and explain the six rating dimensions below, illustrating them with a previously discussed (see Sect. 1.3) example metaphor: supporting an argument.

1. **Accurate**: how accurately the target concept is conveyed;
2. **Complete**: how completely the target concept is conveyed.

The dimensions *accurate* and *complete* are designed to assess general metaphor quality, specifically that of a metaphor’s mappings between its target concept and source domain. Considering our example metaphor of supporting an argument, we might say that it accurately conveys the role of evidence in argumentation, but lacks details of how we find and select that evidence.

3. **Explainable**: how readily the metaphor can be explained by an educator to a learner;
4. **Understandable**: how readily the metaphor can be understood by a learner.

Further, the dimensions *explainable* and *understandable* are intended to assess the pedagogical quality of metaphors for both educators and learners respectively, accounting for the different ways in which either party uses metaphors in an educational setting. Again, looking at our example metaphor, we could say that the notion of support is generally straightforward to explain and understand, regardless of familiarity with writing arguments.

5. **Relatable**: to what extent the metaphor draws upon familiar, tangible experiences;
6. **Actionable**: to what extent the metaphor conveys possible actions and the purpose of these.

Finally, the dimensions *relatable* and *actionable* are designed to assess metaphors’ pedagogical quality in a discipline-specific manner. *Relatable* refers to whether, and how much, a metaphor’s source domain draws upon familiar and tangible experiences—a quality considered to be crucial in supporting science education [51] and also proposed as beneficial for computational thinking [36, 72]. *Actionable* denotes the extent to which a metaphor conveys possible actions related to its target concept, the purpose of these actions, and the consequences of them. This relates to the use of metaphor in computer science [47, 71] and programming [63, 65], in which metaphors’ utility comes from their ability to afford useful actions towards a computing goal. If we again consider our example metaphor, the physical experience of supporting something is both familiar and tangible. Supporting is itself an action, and we can infer that we are supporting an argument *against* something else—but the reason for doing so is still unclear.

In sum, we designed the six rating dimensions to capture how well metaphors might support the learning of scientific concepts, computing practices, and computational thinking skills: key learning outcomes for quantum computing novices with a computing background (as discussed in Sect. 1). As with our first round of expert sessions, we obtained participants’ informed, written consent to audio record, transcribe, anonymise and publish quotes from our discussions with them.

### Metaphor refinement and model synthesis

Following the expert feedback sessions, we conducted a second round of metaphor identification. Incorporating our feedback session participants’ reflections, we re-evaluated the proposed source-target mappings for the initial metaphor set, and re-examined the exploratory interview transcripts for any further metaphors not identified in our initial collection. In doing so, we took a reflexive, iterative and expert-informed approach to metaphor identification, as outlined in Schmitt’s [43] framework.

Having arrived at a final set of metaphors and source-target mappings, we then synthesised a number of metaphorical models, again according to Schmitt’s [43] guidelines. We did so by compiling metaphors with matching target concepts and source domains, and formulating suitable descriptions for each compilation, i.e. a model summary. These model summaries were discussed and iterated upon within the research team until all identified metaphors had been accounted for. In producing these metaphorical models, we aimed to identify the overarching ideas and mechanisms that quantum computing educators draw upon when explaining concepts metaphorically. We also aimed to determine if any of the models were mere commonly used, richer in content, or had greater potential to support quantum computational thinking, based on the quantity and content of the metaphors they contained.

## Results and discussion

### Participants

#### Expert interviews

For our expert interview sessions, we recruited a total of seven participants. All of our participants held current scientific research positions in academia, related to various aspects of quantum computing—with the exception of participant 7, who held expertise in both quantum computing and science communication for quantum technologies. Table 1 provides an overview of the participants’ roles and specific areas of expertise.

**Table 1:** Overview of expert interview participants

Participant	Role	Expertise
P1-I	PhD candidate	Experimental physics (superconducting qubits/single-qubit gates)
P2-I	PhD candidate	Computer science (quantum software development)
P3-I	Postdoctoral researcher	Experimental physics (ion traps for quantum computing)
P4-I	PhD candidate	Experimental physics (superconducting qubits)
P5-I	PhD candidate	Experimental physics (spin qubits)
P6-I	PhD candidate	Hardware engineering (measurement fidelity)
P7-I	Assistant professor	Science communication (quantum technologies), experimental physics (quantum error correction)

#### Expert metaphor feedback sessions

For our expert metaphor feedback sessions, we also recruited a total of seven participants. These participants were also all currently working in academic research positions; five of the seven exclusively held quantum computing-related expertise, whereas participant 4 held expertise in science communication for quantum technologies, and participant 7 in both quantum computing and science communication topics. Table 2 gives an overview of the participants’ roles and areas of expertise.

**Table 2:** Overview of expert feedback session participants

Participant	Role	Expertise
P1-R^*^	PhD candidate	Experimental physics (superconducting qubits)
P2-R	PhD candidate	Experimental physics (superconducting qubits)
P3-R	Postdoctoral researcher	Experimental physics (particle physics for quantum computing)
P4-R	Postdoctoral researcher	Science communication (quantum technologies)
P5-R^*^	PhD candidate	Hardware engineering (measurement fidelity)
P6-R	Postdoctoral researcher	Experimental physics (ion traps for quantum computing)
P7-R	PhD candidate	Science communication (quantum technologies), experimental physics (quantum memory)

^*^Participants marked with an asterisk also took part in our expert interviews.

Although our recruitment criteria included all disciplinary backgrounds related to quantum computing (such as mathematics and computer science, for example), we noted that across both groups of participants, most had expertise in physics-related topics. Our participants are thus fairly representative of the current educational landscape in quantum computing (see Sect. 1.1), but their backgrounds may well have influenced the nature of metaphors and feedback we collected from them.

### Metaphors for QC concepts

Across the expert interview transcripts, we conducted multiple rounds of analysis to collect and refine metaphors related to quantum computing concepts. Through our analysis we identified metaphors for the concepts *quantum algorithms*, *superposition*, *entanglement* and *qubits*. All of the seven participants’ transcripts contained at least one such metaphor, although the number of metaphors identified per participant varied from ten (P5-I) to just one (P6-I), and some of the identified metaphors used the same source-target mapping.

In our first round of analysis, we identified nine initial metaphors for quantum computing concepts. These nine initial metaphors were then presented to participants during our expert feedback sessions. Incorporating our participants’ interpretations, we subsequently enriched and refined our metaphor collection to arrive at a total of 39 distinct metaphors. One third of these pertained to *quantum algorithms* (13/39), followed by *superposition* (12/39), *entanglement* (11/39) and *qubits* (3/39). The Appendix provides a complete overview of the identified metaphors, including the progression from our initial set of nine metaphors to our final set of 39.

**Figure 2:**
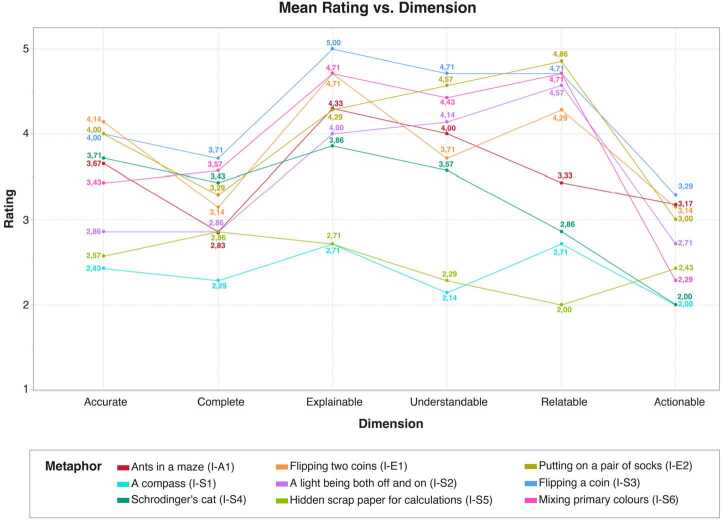
Mean scores given by our feedback session participants to each of the nine initial metaphors, across our six chosen dimensions of quality

During our expert feedback sessions, we asked our participants to reflect on the source-target mappings of the nine initial metaphors, and to score the metaphors along six dimensions of quality (see Fig. 2). A number of patterns in the participants’ comments and scores emerged across these sessions, providing insight regarding the metaphors’ source-target mappings, pedagogical value, and potential to support quantum computational thinking.

#### Source-target mappings

During the expert feedback sessions, we invited participants to reflect on our proposed source-target mappings for the initial nine metaphors. For each metaphor, we asked the participants to indicate whether they agreed with our categorisation of the target QC concept, and to share any alternative or additional target QC concepts they felt were conveyed by the metaphor. Our feedback session participants suggested alternative and/or additional target QC concepts for all nine of the initial metaphors we presented. Not every participant made such suggestions, and the suggestions differed in quantity and content per metaphor. (Given the participants’ varying scientific backgrounds, and the influence of prior experiences on metaphor interpretation [43, 51], such differences are, to an extent, expected.) Despite these differences, some commonalities were apparent across the metaphor set.

Our participants identified *measurement* as a target concept in a number of the metaphors—specifically in those relating to superposition and entanglement. For the entanglement metaphors flipping two coins (I-E1) and putting on a pair of socks (I-E2), and the superposition metaphors a light being both off and on (I-S2), Schrodinger’s cat (I-S4) and mixing primary colours (I-S6), at least one participant suggested measurement as an additional target concept. For example, referring to metaphor I-S4, participant P7-R explained: *“If you perform a measurement, you look into the box and find the cat either dead or alive.”*; similarly, participant P1-R commented, *“It kind of conveys the sense that something is fundamentally uncertain until it’s measured.”*, whilst participant P5-R noted, *“I think what this metaphor is good at, is describing measurement. You have these two ground states, and once you measure, you collapse into one of them.”* However, very few participants referred to specific aspects of the metaphor in question that they felt conveyed measurement. In fact, some participants proposed that measurement, whilst a relevant concept to the metaphor, was actually obscured by the metaphor itself. Referring to metaphor I-E1, participant P5-R commented that, whilst *“there’s measurement involved”*, one would *“need to look for all the parts”* in order to recognise it. Similarly, regarding metaphor I-S6, participant P3-R asserted that *“it fails to mention all these measurement things”*. Additionally, participant P7-R commented that, although metaphor I-E2 could well relate to measurement, they felt that an explanation of *“as the measurer, your influence on the outcome [of measurement]”* was *“lacking”*.

From these reflections, it seems that the concept of measurement (in a quantum computing context) is connected to superposition and entanglement in a significant way, and that several of the metaphors pertaining to superposition or entanglement may thus be interpreted as also conveying measurement. However, the specific mechanisms by which these metaphors actually achieve such conveyance are not always clear—or, rather, they are not acknowledged within the metaphor’s narrative. Whilst ambiguity in metaphor may be considered an inherent feature [73, 74], and in fact has been characterised by some researchers as advantageous for learning purposes [75], here it could lead to a theoretically important concept being missed. Additionally, the framing of measurement in these metaphors seems to be primarily quantum mechanical, with participants using physics terminology such as *“ground states”* (P5-R), *“wave function collapse”* (P3-R) and *“measurement basis”* (P7-R) when describing how measurement was conveyed. Although the participants’ own scientific backgrounds may well have influenced their choice of terminology, it nonetheless seems that these metaphors require a degree of quantum mechanical knowledge to be fully interpreted. This raises questions regarding the metaphors’ educational value—both for learners in general and for those with a specific computing focus. We explore these aspects further in the following section, drawing upon our participants’ rating data and reflections.

#### Pedagogical quality

The feedback session participants’ scores provided insight regarding the initial nine metaphors’ pedagogical quality, as a means of both learning about quantum computing concepts, and of developing quantum computational thinking skills. Figure 2 provides an overview of these scores per metaphor.

Across the initial metaphors, the dimensions *explainable*, *understandable* and *relatable* were scored most highly (with average scores of 4.01, 3.71 and 3.79 respectively), whilst the dimension *actionable* was scored the lowest (average 2.64). The average scores of the dimensions *accurate* and *complete* fell in the middle of this spectrum (3.38 and 3.06 respectively). The average variance of scores per dimension followed a similar pattern, with scores for *explainable* and *understandable* having the least average variance (0.67 and 0.56 respectively), and *actionable* having the highest (1.20). However, the average variance of the dimension *relatable* was comparatively high (0.93), and closer in value to those of the dimensions *accurate* and *complete* (0.91 and 0.86 respectively).

These results suggest that the participants found the initial metaphors to be relatively explainable and understandable, and that there was some agreement amongst the participants regarding what constitutes an explainable or understandable metaphor. Conversely, we can also conclude that the participants considered the initial metaphors minimally *actionable*, but that their perceptions of what makes a metaphor actionable were more varied—as were their opinions on whether a metaphor was *relatable*, *accurate*, and *complete*. These differences might be explained by the variety of the participants’ scientific backgrounds, and consequently their frames of reference for such criteria. For example, participant P3-R found the algorithms metaphor ants in a maze (I-A1) to be inaccurate as it contradicted what they considered “*canonical examples of quantum computing, like Grover’s search algorithm*”, and added that “*we use this same analogy in particle physics, but for very rare cases*”; conversely, participant P2-R described the same metaphor as fairly accurate, with the caveat that they considered themselves “*not an expert [in algorithms]*”. In contrast, the superposition metaphor mixing primary colours (I-S6) was scored as equally *complete* by participants P1-R and P3-R, but their explanations for the score made reference to *“combining binary states”* versus a *“wave function”*: two quite different scientific approaches to the concept of superposition. Such differences highlight the influence of prior knowledge and experience on how individuals interpret a metaphor—even when the metaphor’s target concept is considered fundamental or basic by its community of use, as with superposition [1, 76, 77].

Considerations of prior knowledge were also raised by our participants when discussing the dimensions *explainable* and *understandable*, and the relationship between the two. Participants tended to give similar scores for the dimensions *explainable* and*understandable* per metaphor, although the latter score was, on average, slightly lower. Some participants commented on this tendency directly: referring to the entanglement metaphor flipping two coins (I-E1), participant P3-R described it as *“very explainable”* but *“a bit counter-intuitive, so it’s more difficult to understand, like any analogies with entanglement”*; regarding the same metaphor, participant P6-R considered it highly explainable but thought that *“the twist in saying your coin is tails and mine is heads, that’s adding something that makes it more difficult to understand.”* Similarly, participant P1-R commented that the superposition metaphor a compass (I-S1) *“conveys well how scientists talk about this stuff and what we use as common visualisations”*, but could *“see how it might take some effort to really understand what point is coming across”*. These reflections indicate that, although the metaphors can be useful in teaching and learning their target concepts, some aspects of those target concepts are not clearly explicated within the metaphors’ logic. Without prior knowledge of their target concepts—a likely situation for learners with computing backgrounds, as previously discussed—these metaphors require a logical leap of faith. Such leaps are not uncommon in quantum science communication [78], but are potentially detrimental to broadening participation in the development of quantum technologies [79]. One possible solution proposed in the literature is to frame quantum technologies in terms of information theory and technological implications [78–80]—an approach that, if applied to the metaphors we gathered, could potentially improve their pedagogical value for computing-focused audiences.

As previously discussed, the dimension *relatable* received the second-highest scores on average (3.79), with over half (5/9) of the initial metaphor set receiving average scores of 4 or higher, and three receiving average scores of 4.7 or more. These three metaphors (I-E2, I-S3 and I-S6) involve source domains with tangible and relatively commonplace imagery: a pair of socks, flipping coins and mixing colours. Drawing upon common experiences is considered a characteristic of successful metaphors [40, 57, 58], and the importance of using metaphors that deal with ‘embodied’ phenomena—things that are observable in the world around us—is particularly emphasised in science education [51], with recent work linking embodied metaphors to fostering computational thinking skills [36, 38, 72] (as earlier discussed in Sect. 1). The source domains of the most *relatable* metaphors in our initial set could therefore be fruitful ground for developing metaphors that support quantum computational thinking.

However, metaphors must also be *actionable* in order to be useful for computing. Our initial set of metaphors generally performed poorly in this regard: even the most *explainable*, *understandable* or *relatable* metaphors in our initial set were not considered especially *actionable* by our participants. Some of the metaphors do involve a form of action—flipping a coin (I-S3), putting on a pair of socks (I-E2), or mixing primary colours (I-S6), for example. However, as discussed in the computing metaphors literature (e.g. [47, 63, 65]), a metaphor that illustrates action without application—or possible system response—is limited in its ability to support computing practice. The action must be contextualised: who does it, for what purpose, who else (including the computing system itself) might respond to it, and in what way. Looking at the gathered metaphors, we can see that the actions they contain are rarely given such context. In metaphor I-S6, for example, the purpose of mixing colours is presented as simply creating a visual effect. Similarly, although there are certainly good reasons to put on socks as in metaphor I-E2, the metaphor does not address them and focuses instead on the socks’ physical properties (pairing and sidedness). Whilst the use of tangible, commonplace features can aid metaphors’ conveyance of scientific concepts [51], for computing education, this alone is insufficient. In contrast, the metaphor ants in a maze (I-A1) incorporates both motivation (finding a path through the maze) and system response (movement of ants). Although this metaphor received comparatively low scores for relatability (average 3.43), it was also rated as one of the most actionable (average 3.17). A balance may therefore be needed between the relatability of a metaphor’s source domain, and that source domain’s capacity for purposeful, consequential (inter)actions.

### Metaphorical models for QC concepts

#### Synthesising metaphorical models

Following Schmitt’s Systematic Metaphor Analysis procedure [43] (see Sect. 2), we collated metaphors with the same source and target domains. Over several rounds of iteration—including input from the participants of our expert feedback sessions—we refined these collections to form a number of metaphorical models [40, 43]. In total we formulated 14 metaphorical models, the largest proportion of which pertained to quantum algorithms (5 models), followed by entanglement (4 models), superposition (3 models), and qubits (2 models). Tables 3-6 provide an overview of these metaphorical models, organised by target concept; the Appendix provides details of the metaphors contained in each model.

**Table 3:** Metaphorical models for **quantum algorithms**

Model summary	Example quote	NI	NM	NP
Incremental construction	*You’re building up towards the right answer.*	6	3	4
Applying force towards a desired endpoint	*So you can drive your result, or you want to drive your probability distribution, in a way that you get the right result when you measure it.*	3	3	2
Profiting from a third party	*In a sense you’re taking advantage of Mother Nature. If you ask her to do useful math, you can turn that into a profit.*	3	3	1
Methodical, non-linear movement	*Algorithms are very explorative. It’s meandering, but in the sense that there is a method to that meandering.*	3	3	1
Finding the fastest path	*You try out a bunch of different paths all at the same time, and then you figure out which one works.*	3	1	3

**NI** = number of instances of metaphor use pertaining to model; **NM** = number of distinct metaphors within model; **NP** = number of expert interview participants who employed the model.

**Table 4:** Metaphorical models for **entanglement**

Model summary	Example quote	NI	NM	NP
Paired objects	*You can think of entanglement like a pair of socks.*	6	3	4
Inverse correlation	*When you put a sock on your left foot, automatically, the other sock becomes your right sock.*	5	3	4
Forming couples	*Like couples, coupled to each other.*	3	3	3
Talking	*With entanglement, I would say that you basically get them talking.*	3	2	1

**NI** = number of instances of metaphor use pertaining to model; **NM** = number of distinct metaphors within model; **NP** = number of expert interview participants who employed the model.

**Table 5:** Metaphorical models for **qubits**

Model summary	Example quote	NI	NM	NP
A polar object	*If you have a compass, the natural state is it wants to point towards north. And then the analogy for the kind of excited state is that if you very precisely were to turn it around, such that it exactly pointed the opposite way, it would stick there. And then that’s kind of your other state that would be stable.*	2	2	2
Conversation partners	*Well, you have qubits. They don’t really talk to each other, right? You need to connect them.*	2	1	1

**NI** = number of instances of metaphor use pertaining to model; **NM** = number of distinct metaphors within model; **NP** = number of expert interview participants who employed the model.

**Table 6:** Metaphorical models for **superposition**

Model summary	Example quote	NI	NM	NP
Expansion of resources	*What quantum computing gives you access to is somehow like two to the power of 100 scrap pages...*	7	4	3
Combining orthogonal states	*So you can see a superposition as being in two states at the same time. It’s like a light that’s on and off at the same time.*	6	4	4
Not looking	*...scrap pages that Mother Nature has somewhere, but you cannot see what’s written on them.*	5	4	3

**NI** = number of instances of metaphor use pertaining to model; **NM** = number of distinct metaphors within model; **NP** = number of expert interview participants who employed the model.

#### Model prevalence and robustness

Comparing the prevalence of metaphors and metaphorical models per QC concept, some notable differences emerge. The concept of superposition, for example, was relatively prevalent in our collected metaphors (12/39), but these metaphors led to a relatively small number of metaphorical models (3/14). In contrast, the numbers of both metaphors and metaphorical models for qubits were relatively low (3/39 and 2/14, respectively).

Having a higher number of metaphors versus a lower number of models (for a given concept) can be interpreted as an indicator of how established the models are in their community of use [43]. If a relatively high number of metaphors can be categorised according to a relatively low number of models, this indicates that there is a degree of consensus within the community of use regarding how the target concept is conceptualised metaphorically. Given this, our results suggest that metaphors for superposition may be more plentiful amongst QC educators, and that there is greater agreement amongst these metaphors in terms of their target-source mappings. In other words, there appear to be more clearly defined metaphorical approaches to superposition than to the other QC concepts we identified.

At the other end of the spectrum, we found far fewer metaphors for qubits (3/39) and almost the same number of corresponding models (2/14). Given that all but one of our interview participants explicitly mentioned qubits in their transcripts, it is striking that so few used metaphors to convey them, in comparison to the other QC concepts discussed. With so few metaphors it is challenging to formulate robust metaphorical models; nonetheless, we identified two distinct metaphors for qubits with matching source-target mappings, and a tentative corresponding model (*a polar object*). An additional metaphor was also identified, with two instances of use, albeit by the same participant. Although we propose a possible model for this metaphor (*conversation partners*), further data collection is needed to determine its validity.

One explanation for this lack of qubit-related metaphors may be the way in which our participants framed qubits relative to other QC concepts. In a number of the metaphorical models identified for superposition and entanglement, qubits were implicitly presented as a neutral object, upon which the target quantum concept was projected and thus demonstrated. For example, in conceptualising entanglement as *paired objects*, the notion of *pairing* cannot be demonstrated without *objects to be paired*. In describing superposition as *combining orthogonal states*, this necessitates an *entity whose state can be defined orthogonally*. In this way, the concept of a qubit is embedded in these metaphorical models for other QC concepts, and is in fact an essential component of their logic—but one that is not clearly explicated, and whose properties or use are therefore not fully conveyed.

#### Actionability, embodiment and scale

As previously discussed, actionability and embodiment are critical factors in a metaphor’s ability to support science and computing education, and to foster computational thinking skills. Across the metaphorical models, we identified patterns in their embodiment and actionability, the relative scale of their source domains, and potential consequences for supporting quantum computational thinking.

The metaphorical models we identified for quantum algorithms were all centred around some form of intentional, goal-oriented action: construction, applying force, and pursuing profit, amongst others. A number of the models incorporated movement as a means of progressing towards a desired outcome, although the nature of that progression varied. *Finding the fastest path*, for example, suggests that movement is quick and direct, whereas *applying force towards a desired endpoint* gives a sense of sustained movement, and *methodical, non-linear movement* indicates a slower and perhaps more expansive approach. Each of these imply possible characteristics—efficient, malleable, thorough—that quantum algorithms can possess, and suggest how one might utilise these characteristics when interacting with such algorithms. In this way, the models for quantum algorithms are relatively actionable and thus potentially more useful for computing-focused learners. However, these models are lacking in embodied source imagery; the *subject* of an action is often left abstract, such as *“the right answer”* or a *“probability distribution”*, and with few observable qualities. Given the importance of embodiment in conveying scientific concepts through metaphor (see Sect. 1), it is possible that the models for quantum algorithms may not clearly demonstrate their quantum nature—or at least in a way that is apparent to non-physicists. In contrast, the metaphorical models we present for superposition and entanglement draw upon more embodied, concrete source domains. Familiar physical objects and experiences are commonly used in these models, with some observable properties (such as quantity or directionality) providing a means of illustrating quantum effects. However, conversely to the models for quantum algorithms, they lack the breadth of possible actions—and depth of consequences—that are needed to convey computational relevance. In a sense, the contrast between these models can be described as a matter of scale. Models for quantum algorithms appear to favour overarching procedures in lieu of specific steps, whereas models for superposition and entanglement focus in on one such step—and in doing so, divorce it from its procedural role. For computing-centred learners, an intermediate scale of metaphor—retaining proximity to the goals and processes of algorithms, whilst integrating quantum mechanical aspects contextually—could strike a better balance.

### Developing metaphors to support quantum computational thinking

In collecting and analysing the metaphors presented in this work, we have identified various aspects of metaphor that can either help or hinder learners in developing quantum computational thinking, which we summarise below. We subsequently propose directions for (re)developing metaphors that may better support learners in this regard.

Firstly, we have observed that a number of the metaphors and metaphorical models in our collection make use of embedded yet unacknowledged concepts. As discussed above (see Sect. 3.2.1), the concept of *measurement* is tacitly present in a number of metaphors whose explicit target concept is *superposition* or *entanglement*; similarly, several metaphorical models for these same two target concepts implicitly involve *qubits*. In both cases, the tacit concepts of *measurement* and *qubits* are integral to the metaphors’ logic, but their representations in the metaphors’ source domains are either obscure or simply preconditions for the demonstration of the target concept. The use of tacit information in metaphors, specifically in quantum physics education, is a documented phenomenon that arises from the metaphors’ community of use (i.e. quantum physicists) encoding a “more deep and complex piece of knowledge” within them [52]—in this case, knowledge of how superposition and entanglement are connected to measurement and qubits. Learners, lacking this deeper knowledge, are consequently likely to be confused or misled when encountering such metaphors [52]. For learners with a computing background, additional issues may arise: given that computer science is concerned with “interaction patterns”, as opposed to empirical sciences’ focus on “explanations of observed phenomena” [81], metaphors that obscure the interactions between target concepts can not only be confusing, but counterproductive. When developing metaphors to support quantum computational thinking, rather than taking an “atomic” [71] approach and presenting concepts in isolation, we should instead acknowledge their connections and interdependencies.

In a similar vein, we found that several metaphors relied on logical leaps of faith to accommodate quantum mechanical concepts in physical, real-world source domains—potentially to the detriment of learners’ understanding, and of the metaphors’ computational applicability. Expanding upon prior suggestions in the literature [78–80], we propose that metaphors should be developed to focus on the computational functions and implications of quantum mechanical concepts. This might be achieved by selecting source domains that afford multiple possible actions, but provide clear motivations for, and tangible consequences of them.

Finally, we should be cognisant of the need to balance embodiment and actionability, and how this may relate to the scale of imagery in a chosen source domain. As explored in the previous section, we found that metaphorical models for quantum algorithms tended to operate at the level of a process overview, whereas models for superposition and entanglement typically constrained their scope to a single event. This contrast was reflected in the models’ respective degrees of actionability and embodiment, with algorithm models involving more goal-oriented action but lacking concrete source imagery, and both superposition and entanglement models exhibiting the inverse. These patterns may partially be explained by disciplinary affiliations with the target concepts involved: algorithms are considered to be “computational procedures” or a means to solve a “computational problem” [82], whereas superposition and entanglement are often described in terms of a single physical system [1]. However, engaging with quantum computing as a computing paradigm requires something in between, such that we can “think physically about computation” [1]. Developing new metaphors and metaphorical models can help us to situate and navigate this intermediate space.

To summarise and illustrate, we can once again consider the act of writing. A quantum algorithm might be thought of as a sentence, and superposition, entanglement, or other quantum mechanical concepts as descriptive words. To convey the intended message of our sentence, we must understand which kinds of descriptors are appropriate, but also how to implement them within grammatical conventions: conjugation, word order, and clauses, for example. Grammatical conventions not only help us to produce sentences, but read them, too. An unfamiliar word can still be recognised as an adjective by its placement before a noun—information that allows us to contextually infer its meaning, and perhaps use it in our own sentences if we wish to express something similar. We propose that this intermediate level of grammar encapsulates what metaphors can and should do to support quantum computational thinking: providing patterns and rules that afford action, are applied towards algorithmic goals, and give contextual, functional relevance to unfamiliar quantum mechanical concepts.

### Limitations and future work

This paper contributes a range of quantum computing metaphors and metaphorical models, a framework for their assessment, and suggestions for how they can be further developed to support quantum computational thinking. However, we recognise that our work is an initial exploration of this space and consequently is subject to a number of limitations. Regarding our interview and rating participants, we note that (as discussed earlier in this section), because quantum computing expertise is concentrated in physics institutions, our participants likely brought a quantum mechanical framing to their contributions and ratings of metaphors. We thus aim to complement our current work with a larger and more diverse sample of participants. The inclusion of more algorithm specialists and industry-based experts would be especially beneficial in this regard. We also acknowledge the inherent subjectivity involved in metaphor identification and analysis: our interpretations of the collected metaphors’ target concepts and source domains are our own, and there may well be other or conflicting interpretations that are equally compelling. However, given the lack of prior work exploring metaphors for quantum computing—particularly from a computing-focused perspective—documented analysis of this nature is scarce. As such, we have attempted to follow best practices from the metaphor analysis literature, so that our collection and analysis processes are as replicable as is possible with interpretive work, and that others may build upon the contributions we present here. In any case, developing metaphors is only part of the puzzle: if we are indeed able to produce metaphors that have potential to support quantum computational thinking, we cannot verify this potential without implementing the metaphors in education. Future work in this area should therefore explore the use of such metaphors in designing educational materials and interventions.

## Conclusion

In this paper, we set out to gather metaphors used by quantum computing experts in educational contexts, to explore possible overarching metaphorical models, and to assess the pedagogical value of these metaphors and models for learning about quantum computing concepts, supporting computer science practices, and developing computational thinking skills. Based on the above, we aimed to suggest how such metaphors might better facilitate quantum computational thinking.

Through an iterative process of identifying, interpreting and categorising, we gathered a total of 39 distinct metaphors for four quantum computing concepts: quantum algorithms, superposition, entanglement, and qubits. From these 39 metaphors, we propose 14 metaphorical models that describe common source-target mappings amongst the metaphors. We find that metaphors and metaphorical models for qubits are relatively scarce, arguing that this stems in part from the framing of qubits as a precondition for quantum mechanical effects.

Based on our participants’ feedback on an initial set of nine metaphors, and our own further analysis of the complete metaphor set, we offer the following insights and suggestions:

1. The participants’ reflections indicate that the gathered metaphors may be less comprehensible for learners than for educators, and that the metaphors tend to rely on logical leaps of faith—presumably intended to circumvent counter-intuitive aspects, but potentially confusing learners or obscuring important concepts. We suggest that metaphors should instead focus on the computational functions and implications of a concept, and in doing so can better accommodate otherwise counter-intuitive aspects.
2. Additionally, we find that the gathered metaphors often appear designed to conceal interdependencies between their target concepts and others. We argue that such interdependencies are in fact helpful when approaching quantum concepts computationally, and that metaphors should thus acknowledge their relatedness.
3. Finally, we conclude that the gathered metaphors lack actionability, and therefore have limited relevance for computing-centred learning. We propose that metaphors should be developed with greater emphasis on the application and implications of their target concepts, using source domains that balance procedural narratives with embodied imagery.

The quantum computing landscape is constantly evolving, and it is possible that future end users of this technology may engage with it purely as a source of processing power. In such a scenario, the kinds of metaphors we present in this paper may become less relevant. What seems more certain is that computing skills and knowledge are critical to the progress of quantum computing, and that metaphors could help to bring individuals with computing backgrounds into the fold. We thus intend for our work to illustrate how such metaphors might be developed, and hope that it serves as a useful basis for further explorations in this area.

## Data Availability

The expert rating data generated by and analysed in this study is available under a Creative Commons 4.0 license in the 4TU Repository, https://doi.org/10.4121/f225eb54-9bcf-491a-baf9-15a933ccb1d0.v1. The authors confirm that all other data generated and analysed for the purposes of this study are contained within this manuscript.

## Appendix: Collected metaphors for QC concepts

**Table 7:** Initial set of collected metaphors for QC concepts

Code	Target concept	Source domain	Quote
I-A1	Algorithms	Ants in a maze	*Kind of like ants in a maze, you can use not many things to describe a tonne of things. Now imagine if you were able to use that tonne of things as paths in a maze, and you try out a bunch of different paths all at the same time. And then you figure out which one works.*
I-E1	Entanglement	Flipping two coins	*Entanglement is like, I flip a coin and you flip a coin, and every time I get heads, you get tails, and the other way around.*
I-E2	Entanglement	Putting on a pair of socks	*You can think of entanglement like a pair of socks. When you put a sock on your left foot, automatically, the other sock becomes your right sock. So these two socks are entangled.*
I-S1	Superposition	A compass	*If you have a compass, the natural state is it wants to point towards north. And then the analogy for the kind of excited state is that if you very precisely were to turn it around, such that it exactly pointed the opposite way, it would stick there. And then that’s kind of your other state that would be stable.*
I-S2	Superposition	A light being both off and on	*So you can see a superposition as being in two states at the same time. It’s like a light that’s on and off at the same time.*
I-S3	Superposition	Flipping a coin	*For instance, you have a coin. You flip it and if you look at it it’s a definite head or a definite tail, but as long as you don’t look at it, it can be both [heads and tails].*
I-S4	Superposition	Schrodinger’s cat	*What is the meaning of superposition? What actually happens? You must have already heard the example of Schrodinger’s cat. The question, you know, is it dead or alive? Or is it actually in a superposition of both [dead and alive]?*
I-S5	Superposition	Hidden scrap paper for calculations	*What quantum computing gives you access to is somehow like two to the power of 100 scrap pages that Mother Nature has somewhere, but you cannot see what’s written on them. You can only try to use intuition and clever tricks to take advantage of nature’s bookkeeping. And then the art of trying to build a quantum computer is how do you make use of this paper that you can’t write on, and you can’t look at, how do you take advantage of that to solve problems?*
I-S6	Superposition	Mixing primary colours	*Essentially, a superposition is just like a combination of two things. If you think of combining red and blue, you get some shade of purple.*

**Table 8:** Final set of collected metaphors for QC concepts

Code	Target concept	Source domain	Model	Quote	Parent
F-A1	Algorithms	Finding the fastest path	Finding the fastest path	*Now imagine if you were able to use that tonne of things as paths in a maze, and you try out a bunch of different paths all at the same time. And then you figure out which one works.*	I-A1
F-A2	Algorithms	Taking advantage of nature	Profiting from a third party	*You can only try to use intuition and clever tricks to take advantage of nature’s bookkeeping.*	I-S5
F-A3	Algorithms	Asking nature to calculate for you	Profiting from a third party	*If you ask Mother Nature to do useful math, you can turn that into a profit.*	-
F-A4	Algorithms	Taking advantage of hidden paper	Profiting from a third party	*And then the art of trying to build a quantum computer is how do you make use of this paper that you can’t write on, and you can’t look at, how do you take advantage of that to solve problems?*	I-S5
F-A5	Algorithms	Building up an answer	Incremental construction	*So you build up the answer that you want.*	-
F-A6	Algorithms	Building up confidence	Incremental construction	*You cannot just run it once and be satisfied with it. You want to build up confidence.*	-
F-A7	Algorithms	Building blocks	Incremental construction	*The thing in between, like the algorithm, is very huge. And then I think you have to break down what the quantum system looks like, like the two building blocks zero or one in normal classical computing. What does this building look like?*	-
F-A8	Algorithms	Driving a result	Applying force towards a desired endpoint	*So you can drive your result, or you want to drive your probability distribution, in a way that you get the right result when you measure it.*	-
F-A9	Algorithms	Forcing an answer	Applying force towards a desired endpoint	*...so that we force the right answer at the end.*	-
F-A10	Algorithms	Driving towards a point	Applying force towards a desired endpoint	*Our algorithm will kind of drive it towards the zero point.*	-
F-A11	Algorithms	Meandering methodically	Methodical, non-linear movement	*It’s meandering, but in the sense that there is a method to that meandering.*	-
F-A12	Algorithms	Exploring methodically	Methodical, non-linear movement	*Even if it is explorative, we have a method towards that exploration.*	-
F-A13	Algorithms	Dancing around	Methodical, non-linear movement	*How do we, you know, work or dance around this probability distribution?*	-
F-E1	Entanglement	Flipping two coins	Paired objects	*Entanglement is like, I flip a coin and you flip a coin...*	I-E1
F-E2	Entanglement	A pair of socks	Paired objects	*You can think of entanglement like a pair of socks.*	I-E2
F-E3	Entanglement	A pair of shoes	Paired objects	*If someone takes a pair of shoes, shuffles them up, puts one in a box...*	-
F-E4	Entanglement	Heads or tails	Inverse correlation	*Every time I get heads, you get tails, and the other way around.*	I-E1
F-E5	Entanglement	Left or right	Inverse correlation	*When you put a sock on your left foot, automatically, the other sock becomes your right sock.*	I-E2
F-E6	Entanglement	Left or right	Inverse correlation	*...you open up one box and see it’s a left shoe, so you know the other one must be a right shoe.*	-
F-E7	Entanglement	Facilitating conversation	Talking	*With entanglement, I would say that you basically get them talking to each other.*	-
F-E8	Entanglement	Observing conversation	Talking	*With entanglement, they’re all talking somehow.*	-
F-E9	Entanglement	A couple	Forming couples	*Like couples, coupled to each other.*	-
F-E10	Entanglement	Connecting two parties	Forming couples	*We can actually create connection between two parties.*	-
F-E11	Entanglement	Inherent connection	Forming couples	*They are always connected, always, independent of distance or whatever you do with them.*	-
F-Q1	Qubits	A compass	A polar object	*If you have a compass, the natural state is it wants to point towards north. And then the analogy for the kind of excited state is that if you very precisely were to turn it around, such that it exactly pointed the opposite way, it would stick there. And then that’s kind of your other state that would be stable.*	I-S1
F-Q2	Qubits	An object with poles	A polar object	*So if you go from zero to one, that would take you from here to there. So you just switched the pole.*	-
F-Q3	Qubits	Conversation partners	Conversation partners	*Well, you have qubits. They don’t really talk to each other, right? You need to connect them.*	-
F-S1	Superposition	Both on and off	Combining orthogonal states	*So you can see a superposition as being in two states at the same time. It’s like a light that’s on and off at the same time.*	I-S2
F-S2	Superposition	Both heads and tails	Combining orthogonal states	*...it can be both [heads and tails].*	I-S3
F-S3	Superposition	Both dead and alive	Combining orthogonal states	*Or is it actually in a superposition of both [dead and alive]?*	I-S4
F-S4	Superposition	Both blue and red	Combining orthogonal states	*If you think of combining red and blue, you get some shade of purple.*	I-S6
F-S5	Superposition	Exponential description	Expansion of resources	*Kind of like ants in a maze, you can use not many things to describe a tonne of things.*	I-A1
F-S6	Superposition	Exponential scrap paper	Expansion of resources	*What quantum computing gives you access to is somehow like two to the power of 100 scrap pages...*	I-S5
F-S7	Superposition	Greater access and power	Expansion of resources	*With superposition, essentially, you have access to more states, so you have more computing power.*	-
F-S8	Superposition	Exponential space for writing	Expansion of resources	*Superposition gives you this exponential increase in, let’s say, the size of the page you can use.*	-
F-S9	Superposition	Looking away	Not looking	*You flip it and if you look at it it’s a definite head or a definite tail, but as long as you don’t look at it...*	I-S3
F-S10	Superposition	Being unable to observe	Not looking	*The question, you know, is it dead or alive?*	I-S4
F-S11	Superposition	Being unable to see	Not looking	*...pages that Mother Nature has somewhere, but you cannot see what’s written on them.*	I-S5
F-S12	Superposition	Looking away	Not looking	*If I turn around and don’t look at this table any more, is it still there?*	-

## Footnotes

1 Lakoff and Johnson refer to metaphorical models as *metaphorical concepts*, whereas Schmitt uses the term *models* to emphasise their role as cognitive structures. As we draw upon Schmitt’s analytical methodologies in this work, we have aligned our use of terminology accordingly.

## Abbreviations

QC: Quantum Computing
QM: Quantum Mechanics
CT: Computational Thinking
